# An Unusual Presentation of Life-Threatening Necrotising Mediastinitis in an Adolescent

**DOI:** 10.1155/cris/5551040

**Published:** 2025-06-19

**Authors:** Henrik Agerup Kildahl, Anna Greta Birgitta Ehrnstrom, Per Magnus Haram, Geir Bjerkan, Katrine Hordnes Slagsvold, Øystein Pettersen

**Affiliations:** ^1^Department of Cardiothoracic Surgery, St Olavs University Hospital, Trondheim, Trøndelag, Norway; ^2^Department of Circulation and Medical Imaging, Faculty of Medicine and Health Sciences, Norwegian University of Science and Technology, Trondheim, Trøndelag, Norway; ^3^Department of Infection, St Olavs University Hospital, Trondheim, Trøndelag, Norway; ^4^Department of Orthopaedics, Rheumatology and Dermatology, St Olavs University Hospital, Trondheim, Trøndelag, Norway

## Abstract

A male in early adolescence presented with 1 week of chest pain, respiratory symptoms and diarrhoea. Thoracic computed tomography (CT) revealed suspicious findings of necrotising mediastinitis without signs of a descending infection. The patient underwent bilateral thoracotomy and laparotomy with several revisions. After 58 days in the hospital, the patient was discharged home, fully recovered, with no sequelae. This case highlights the importance of a multidisciplinary approach when managing severe and rare conditions, emphasising the need for early diagnosis and prompt, appropriate surgical treatment.

## 1. Introduction

Necrotising mediastinitis is a rare clinical entity with limited available literature. Existing case reports refer to necrotising mediastinitis due to a descending infection from the oral cavity or neck or as a complication of a previous intervention [1]. The disease carries high morbidity and mortality [1, 2], with septic shock as a predictor of mortality [3]. There is currently no established treatment other than rapid antimicrobial treatment and surgical debridement [3, 4]. There are no guidelines available and only sparse literature and case reports [2, 5, 6].

## 2. Case Report

A presumed healthy young adolescent male presented to our tertiary referral hospital after experiencing 5–6 days of fever and progressively worsening chest pain. The patient had haemoptysis, dysphagia, severe dyspnoea and diarrhoea. The clinical history revealed a chronic cough over the past months. The vaccination status and past medical history of the patient were unknown.

**Figure 1:**
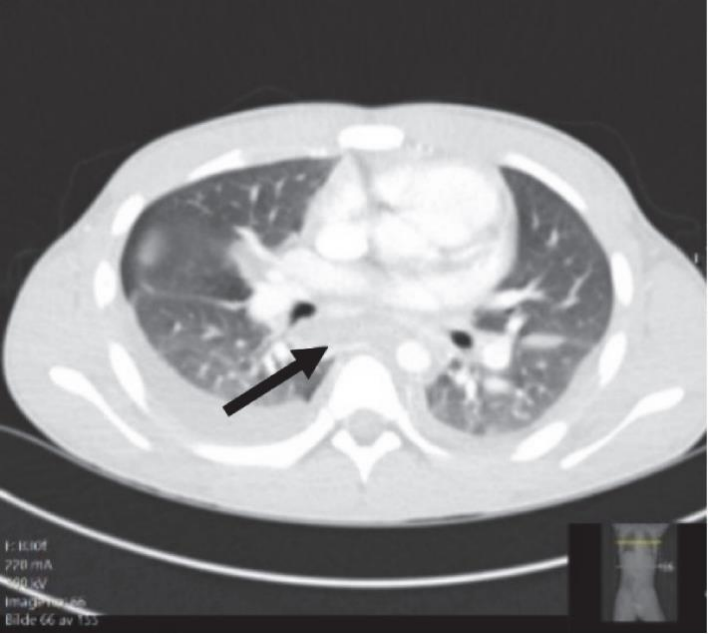
CT performed upon admission; arrow indicates increased soft tissue in the mediastinum.

Vital parameters upon admission were blood pressure 80/40 mmHg, heart rate 120 bpm, respiratory rate of 40 per minute and saturation 96% on room air. Physical examination revealed an itching maculopapular rash on the trunk and extremities, dry mucous membranes and tender cervical lymphadenopathy. The physical examination was consistent with a severe respiratory infection, but no signs were indicative of necrotising fasciitis. A computed tomography (CT) scan was done upon admission (Figure 1), showing bilateral pleural effusion, ground glass opacities and a widened mediastinum.

Blood cultures were positive for *Streptococcus pyogenes* (*group A streptococci*). The patient required vasopressors to maintain a median arterial pressure (MAP) above 65 mmHg. He had an elevated lactate despite volume resuscitation and fulfilled the criteria for septic shock with signs of multiorgan failure. The patient was intubated and put on a ventilator after <12 h at the intensive care unit.

**Figure 2:**
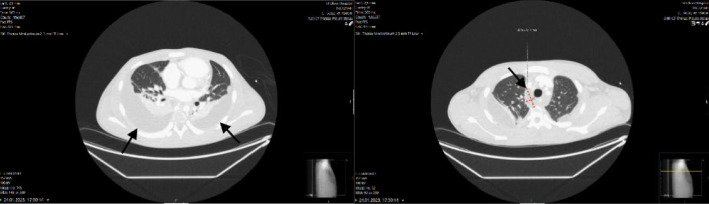
CT showed increased pleural effusions and fluid in the mediastinum.

A second CT (Figure 2) was done on day 2 after admission (due to no clinical improvement despite broad antimicrobial coverage). The CT revealed signs indicative of bilateral empyema and increasing fluid accumulation apically in the mid mediastinum, dorsal to the oesophagus and surrounding the aorta. It was specifically mentioned that imaging did not identify any signs of a descending abscess from the oropharynx to the mediastinum. The CT findings were suspicious of an infectious mediastinitis.

On the third day of admission, 32-sized French chest drains were inserted bilaterally into the pleura and drained 900 mL of serous fluid on the right side, and 450 mL from the left side. A brief clinical improvement followed with a slight reduction in doses of vasoactive medications and a discrete drop in CRP.

**Figure 3:**
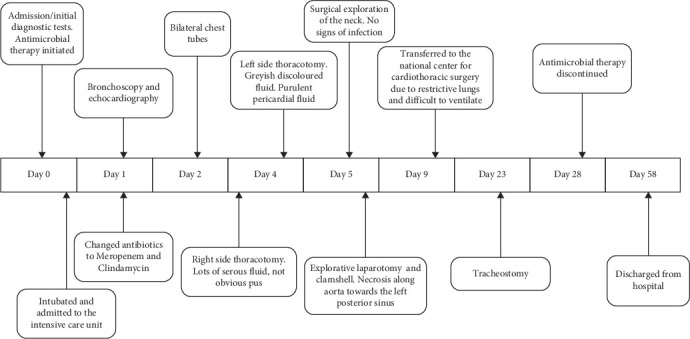
Timeline of events during the admission.

A right-sided thoracotomy with extensive revision of the chest cavity was performed early on the fourth day after admission. A thorough exploration of lymph stations between vena cava superior, vena cava posterior towards the ascending aorta and laterally towards the trachea, in addition to below the carina and posterior to the trachea. Samples were sent for both culture and microscopy. Direct microscopy showed abundant cocci in chains, typical for *S. pyogenes*.  Figure 3 displays a timeline of major events during the admission (Figure 3).

There was no significant clinical improvement postoperatively with high doses of vasopressors. The clinical state, combined with findings on the second CT suggestive of infection also in the left thoracic cavity and cocci in chains on direct microscopy, prompted further surgical intervention on the left side. An orthopaedic surgeon with expertise in necrotising fasciitis accompanied this procedure. Experience with necrotising infections and the degree of radicality it demands was paramount to achieve disease control. During this operation, significant amounts of greyish fluid, as well as white fibrin layers and pus were present and extended towards the caudal parts of the aorta and oesophagus. The pericardium, which had a normal surface anatomy, was opened where a purulent pericardial fluid was present.

The patient was still in critical condition on the fifth day after admission. The patient was re-operated with an explorative laparotomy due to necrosis extending along the oesophagus and aorta at the base of the diaphragm. It was done with a simultaneous clamshell procedure and a surgical exploration of the neck. There were no obvious foci for infection in the abdomen, except for necrosis behind the oesophagus at the level of the diaphragm crura, with further extensions of the necrosis cranially into the thoracic cavity along the lateral aorta and further towards the left posterior sinus. Extensive debridement of the thoracic cavity, including all pre- and pericardial subcutaneous tissue and parts of the thymus, was done. An abdominal vacuum-assisted closure (VAC) closed the abdomen. The thoracotomy was closed with a continuous suture. A surgical exploration of the neck revealed no signs of infection.

A second and third look on the sixth and seventh day after admission showed no signs of infection or necrosis. There was a subsequent improvement of the clinical condition and no need for vasopressor 8 days after admission.

On the ninth day after admission, the patient deteriorated with an increasing need for vasopressor, and a significantly reduced lung compliance. The patient still had VAC of the abdomen, and the thorax was temporarily closed with continuous rinse, where saline infusion on one chest drain and evacuated on the other drain [7].

The patient had severely reduced lung compliance and required dangerously high pressures on the respirator to maintain adequate oxygenation. Due to the availability of Prisma Lung, the patient was transferred to the National Centre for Cardiothoracic Surgery, as this could be necessary due to his severely restrictive lungs.

The patient gradually improved, there was no need for Prisma Lung, and the patient was transferred back to our centre. When weaned off the ventilator, he received a tracheostomy temporarily due to high demands on the respirator and one expected prolonged awakening. He was extubated successfully and transferred from the ICU to the regular ward. He required extensive physiotherapy, as well as a clinical nutritionist, during his rehabilitation. There was a gradual and steady improvement in the general condition, and after a further 3 weeks, he was mobile, eating well and discharged home. He had no neurological complications but described pain concerning the laparotomy.

## 3. Comment

There are several learning points from this case report as follows.

• The absence of an oropharyngeal infection focus does not exclude the possibility of necrotising mediastinitis.
• A multidisciplinary approach should be initiated early.
• Direct microscopy as a diagnostic aid can save time and enable rapid targeted antibiotic treatment and surgical debridement.
• Opening of the pericardium, even though normal-looking surface anatomy on the outside, revealed purulent pericardial fluid and obvious signs of infection.

## Data Availability

The clinical data that support the findings in this case report are included in the case report.
